# Mutations in *Rice yellow mottle virus* Polyprotein P2a Involved in *RYMV2* Gene Resistance Breakdown

**DOI:** 10.3389/fpls.2016.01779

**Published:** 2016-11-28

**Authors:** Agnès Pinel-Galzi, Christine Dubreuil-Tranchant, Eugénie Hébrard, Cédric Mariac, Alain Ghesquière, Laurence Albar

**Affiliations:** ^1^Interactions Plantes Microorganismes Environnement, Institut de Recherche pour le Développement – Centre de Coopération Internationale en Recherche Agronomique pour le Développement – Université de MontpellierMontpellier, France; ^2^Plant Diversity Adaptation and Development Research Unit, Institut de Recherche pour le Développement – Université de MontpellierMontpellier, France

**Keywords:** rice, RYMV, CPR5, resistance breakdown, virus adaptation, deep sequencing

## Abstract

*Rice yellow mottle virus* (RYMV) is one of the major diseases of rice in Africa. The high resistance of the *Oryza glaberrima* Tog7291 accession involves a null allele of the *RYMV2* gene, whose ortholog in *Arabidopsis*, *CPR5*, is a transmembrane nucleoporin involved in effector-triggered immunity. To optimize field deployment of the *RYMV2* gene and improve its durability, which is often a weak point in varietal resistance, we analyzed its efficiency toward RYMV isolates representing the genetic diversity of the virus and the molecular basis of resistance breakdown. Tog7291 resistance efficiency was highly variable depending on the isolate used, with infection rates ranging from 0 to 98% of plants. Back-inoculation experiments indicated that infection cases were not due to an incomplete resistance phenotype but to the emergence of resistance-breaking (RB) variants. Interestingly, the capacity of the virus to overcome Tog7291 resistance is associated with a polymorphism at amino-acid 49 of the VPg protein which also affects capacity to overcome the previously studied *RYMV1* resistance gene. This polymorphism appeared to be a main determinant of the emergence of RB variants. It acts independently of the resistance gene and rather reflects inter-species adaptation with potential consequences for the durability of resistance. RB mutations were identified by full-length or partial sequencing of the RYMV genome in infected Tog7291 plants and were validated by directed mutagenesis of an infectious viral clone. We found that Tog7291 resistance breakdown involved mutations in the putative membrane anchor domain of the polyprotein P2a. Although the precise effect of these mutations on rice/RYMV interaction is still unknown, our results offer a new perspective for the understanding of *RYMV2* mediated resistance mechanisms. Interestingly, in the susceptible IR64 variety, RB variants showed low infectivity and frequent reversion to the wild-type genotype, suggesting that Tog7291 resistance breakdown is associated with a major loss of viral fitness in normally susceptible *O. sativa* varieties. Despite the high frequency of resistance breakdown in controlled conditions, this loss of fitness is an encouraging element with regards to *RYMV2* resistance durability.

## Introduction

Varietal selection is generally considered as an effective way to control the impact of plant diseases on crop yield. However, the emergence of pathogen variants that have evolved to overcome plant resistance mechanisms is a major impediment to its use. Depending on the resistance genes, resistance breakdown may occur within few years or decades (Janzac et al., 2009), and resistance durability prediction is thus of main concern for breeders and farmers. In recent years, several retrospective and modeling studies have focused on identifying the main factors involved in resistance durability with the aim of drawing up resistance gene selection guidelines for breeders and resistance gene deployment strategies for farmers. At the field scale, the emergence of virulent variants depends mostly on epidemiological factors: inoculum pressure, transmission mode, vector and reservoir populations, and landscape connectivity (Fabre et al., 2012b). However, the pathogen population first has to evolve in the resistant plant at the intra-host level. At this scale, the major determinants of resistance durability are the genetic changes required for a pathogen to overcome plant resistance mechanisms and the effects of such changes on pathogen fitness (Fabre et al., 2009).

The *rice yellow mottle virus* (RYMV) is a major biotic constraint for rice cultivation in Africa. An in depth analysis of RYMV diversity revealed that the virus emerged 200 years ago in East Africa and dispersed throughout the continent (Pinel-Galzi et al., 2015). Six strains, i.e., genetic groups, have been identified based on molecular and serological analysis. RYMV is now prevalent in almost all Sub-Saharan countries where rice is cultivated and is responsible for major losses in rice varieties adapted to lowland and irrigated ecosystems (Kouassi et al., 2005). Various transmission vectors, including insects and animals, as well as agricultural practices, particularly seedbed to field transplantation, complicate the disease management. Prophylactic measures, such as high surveillance of seedbeds, fields and surrounding wild rice and weed stands to decrease the inoculum pressure, are time-consuming and of variable efficiency. The use of resistant varieties is a highly promising strategy to reduce damage, but resistance durability is critical for sustainable control of the disease.

*RYMV1* is the first resistance gene described in the rice/RYMV interaction. It controls resistance in a recessive way and encodes eIF(iso)4G1, a translation initiation factor (Ndjiondjop et al., 1999; Albar et al., 2006). Accessions carrying a *RYMV1* resistance allele do not develop any symptoms, nor do they show detectable virus content in ELISA serological assays, although residual virus multiplication can be detected by quantitative reverse transcriptase-polymerase chain reaction (Ndjiondjop et al., 2001; Poulicard et al., 2010). Four independent *RYMV1* resistance alleles have been reported in the Asian cultivated rice species *Oryza sativa* (*rymv1-2* allele), or in the African cultivated rice species *Oryza glaberrima* (alleles *rymv1-3*, *-4*, and *-5*) (Albar et al., 2006; Thiémélé et al., 2010). Mutations conferring resistance involved point mutations or small deletions. The mutation characterizing *rymv1-2* impairs the interaction between eIF(iso)4G1 and VPg, a viral protein covalently linked to the viral genome (Hébrard et al., 2010), as observed in the interplay between plant eIF4E translation factors and the VPg of several potyviruses (for review Sanfaçon, 2015). *RYMV1* has only been very recently deployed in the field (Bouet et al., 2013; Ndjiondjop et al., 2013) and its durability is still unknown. However, the emergence of RYMV variants able to overcome the resistance of accessions with *rymv1-2* and *rymv1-3* alleles has been observed in experimental conditions (Fargette et al., 2002; Traoré et al., 2006). Resistance-breaking (RB) mutations arise through a stepwise process in codons of the VPg central domain (Pinel-Galzi et al., 2007; Traoré et al., 2010). Substitutions on codon 48 are the most frequently observed efficient mutations in resistance breakdown in the *O. sativa* Gigante accession, which carries the *rymv1-2* allele, while substitutions on codons 41 and/or 52 are generally involved in resistance breakdown in the *O. glaberrima* Tog5681 accession, which carries the *rymv1-3* allele. Resistance breakdown is associated with restoration of the interaction between the central domain of the resistance gene product and the mutated VPg (Hébrard et al., 2010). Comparison of RB processes in Gigante and Tog5681 accessions revealed similarities in the mode of adaptation, but also revealed a converse genetic barrier associated with the polymorphism at VPg codon 49 (Poulicard et al., 2012). Isolates with a glutamic acid (E49) do not overcome the resistance of Tog5681 (*rymv1-3* allele), and those with a threonine (T49) rarely overcome Gigante (*rymv1-2* allele), although a small subset of T49 isolates from Central Africa is able to overcome Gigante (Traoré et al., 2010). A selective advantage of T49 isolates over E49 isolates in susceptible *O. glaberrima* accessions has been demonstrated and is assumed to explain this contrasted RB ability (Poulicard et al., 2012). Given that only E49 isolates are present in East Africa, while both types of isolate are present in West and Central Africa, the deployment of *O. sativa* high-yielding varieties with the *rymv1-3* resistance allele is promising in East Africa, but additional resistance genes may be necessary to confer durable resistance in other regions.

Resistance sources independent of *RYMV1* have been identified in the *O. glaberrima* species (Thiémélé et al., 2010). Tog7291 has a high resistance, recessively inherited phenotype characterized by the absence of symptoms and a very low virus content, which is generally undetectable via ELISA. Tog7291 resistance involves the *RYMV2* resistance gene, identified as *CPR5-1* (*Constitutive expressor of Pathogenesis Related proteins-5*) (Orjuela et al., 2013). This gene is assumed to encode a transmembrane nucleoporin controlling defense mechanisms, as suggested by the findings of studies on its ortholog from *Arabidopsis thaliana* (Bowling et al., 1997; Wang et al., 2014; Gu et al., 2016). The resistance allele of *CPR5-1* identified in Tog7291 is a null allele, characterized by a 1-bp deletion in the first exon. So far this resistance has been assessed against a single RYMV isolate. Here, we described the ability of isolates representative of the genetic diversity of RYMV to overcome high Tog7291 resistance and we analyzed the molecular basis of resistance breakdown.

## Materials and Methods

### Plant Material

Tog7291 (International Rice Germplasm Collection number 104589) is an *O. glaberrima* accession that is highly resistant to RYMV (Thiémélé et al., 2010) and carries a null allele on the *RYMV2* gene (Orjuela et al., 2013). The *O. sativa* spp. *indica* IR64 variety and *O. glaberrima* Tog5673 (IRGC number 96789) accession were used as susceptible controls.

### RYMV Isolates

The 20 RYMV isolates used in this study are described in **Table 1**. Isolates were classified according to their country of origin, strain and amino acid at position 49 of their VPg. The sampling included five E49 isolates from East Africa, representing strains S4, S5, and S6, and four E49 and 11 T49 isolates from West Africa, representing strains S1 and S2. The isolates were multiplied on the IR64 susceptible variety and stored at -20°C. Inoculum was prepared by grinding infected leaves in 0.1M phosphate buffer pH 7.2 (0.1 g/ml) and adding 600-mesh carborundum.

**Table 1:** Characteristics of the RYMV isolates used in this study.

Name	Country of origin	VPg49	Strain	Reference
BF1	Burkina Faso	T	S2	Pinel et al., 2000
CIa	Côte d’Ivoire	T	S2	Fargette et al., 2004
CI2	Côte d’Ivoire	T	S1	Pinel et al., 2000
CI3	Côte d’Ivoire	T	S1	Pinel et al., 2000
CI8	Côte d’Ivoire	T	S2	Pinel et al., 2000
CI63	Côte d’Ivoire	T	S2	Fargette et al., 2004
CI4	Côte d’Ivoire	E	S1	Pinel et al., 2000
Ni1	Nigeria	T	S1	Pinel et al., 2000
Tg247	Togo	T	S1	This study
Tg274	Togo	T	S1	Pidon et al., submitted
Ng106	Niger	T	S1	Traoré et al., 2010
Ng119	Niger	T	S1	Pidon et al., submitted
Ng109	Niger	E	S1	Traoré et al., 2010
Ng111	Niger	E	S1	Pidon et al., submitted
Ng113	Niger	E	S1	Pidon et al., submitted
Mg1	Madagascar	E	S4	Pinel et al., 2000
Tz5	Tanzania	E	S4	Fargette et al., 2004
Tz11	Tanzania	E	S6	Abubakar et al., 2003
Tz201	Tanzania	E	S4	Fargette et al., 2008
Tz211	Tanzania	E	S5	Fargette et al., 2008


*The country of origin, the amino acid at position 49 of the VPg, and the strain, i.e., genetic group, are indicated for each isolate.*

A new isolate collected in Togo, Tg247, was included in the viral corpus. After total RNA extraction with the RNeasy Plant Mini kit (Qiagen, Germany). The 720 nucleotide coat protein gene was amplified by reverse transcription-polymerase chain reaction (RT-PCR) to determine the viral strain (Fargette et al., 2002). In addition, the VPg and its 5′ and 3′ neighboring regions were sequenced as previously described (Hébrard et al., 2006).

The CIa^∗^49E mutant was obtained by Poulicard et al. (2012) using directed mutagenesis of the infectious clone CIa obtained by Brugidou et al. (1995) to introduce a glutamic acid (E) instead of a threonine (T) at position 49 of the VPg. Additional mutants were constructed in this study by directed mutagenesis of the infectious clone CIa with the QuickChange Site-Directed Mutagenesis Kit (Stratagene), as described in Hébrard et al. (2006). The mutants were generated by the introduction of a glycine (G) instead of E at position 64 of polyprotein P2a (CIa^∗^P2a-64G), of a cysteine (C) or a leucine (L) instead of phenylalanine (F) at position 66 (CIa^∗^P2a-66C and CIa^∗^P2a-66L, respectively), an alanine (A) instead of valine (V) at position 70 (CIa^∗^P2a-70A), and of an L instead of F at position 115 (CIa^∗^P2a-115L). After *in vitro* transcription, the variants were multiplied in IR64.

### Phenotypic Evaluation of Resistance and Resistance Breakdown

The percentage of plants from the Tog7291 accession that were infected was assessed on 28 plants per isolate for isolates CI3, CI63, Ng111, Ng119, Tg247, Tz11, and Tz201 and on 51 to 56 plants for the others. Plants were cultivated in glasshouse conditions in a tray of 28 individual 350 ml pots. Plants were mechanically inoculated about 2 weeks after sowing by rubbing the inoculum on leaves. Symptoms were observed from 2 to 6 weeks after inoculation. Samples from the last emerged leaf were collected 4 weeks after inoculation for all isolates and 6 weeks after inoculation for 12 isolates (BF1, CI4, CI8, Ni1, Ng106, Ng109, Ng113, Tg274, Mg1, Tz5, Tz211). Direct double antibody sandwich enzyme-linked immunosorbent assay (DAS-ELISA) was performed as described by Ndjiondjop et al. (1999) with a polyclonal antiserum prepared against a RYMV isolate from Madagascar (N’Guessan et al., 2000). About 1 mg of fresh leaves in 1 ml buffer was used. Samples were considered negative when the optical density was lower than threefold the optical density of the negative controls. When samples were collected at two dates—considering that recovery from the infected state is highly unlikely—ELISA tests were first performed on samples collected 4 weeks after inoculation on symptomatic plants, and, if negative, on samples collected 6 weeks after inoculation. In the case of non-symptomatic plants, ELISA tests were first performed on samples collected at 6 weeks and, if positive, on samples collected at 4 weeks.

Selected samples from infected Tog7291 plants were tested in back-inoculation assays on up to 10 IR64 and 10 Tog7291 plants. The culture and inoculation conditions were as described above. Symptoms were observed between 2 and 3 weeks after inoculation. ELISA tests were performed 3 weeks after inoculation on plants with no or unclear symptoms.

Two different assessments on the mutants obtained by directed mutagenesis were conducted. In the first experiment, after *in vitro* transcription, the mutants were multiplied once on IR64 and then inoculated on eight IR64 plants and 28 Tog7291 plants, in the same conditions as described above. For the second experiment, the mutants were multiplied a second time on IR64. The obtained inoculum concentration was evaluated by Q-RT-PCR, as described in Poulicard et al. (2010), and adjusted to 2.10^9^ or 3.10^10^ copies of the virus in 100 μl of inoculum. Five plants of accessions IR64, Tog5673, and Tog7291 were inoculated with the mutants or the wild-type control by rubbing 100 μl of inoculum on the leaves of each plant. For both experiments, ELISA tests were performed on all plants 2 weeks after inoculation and samples were collected for Sanger sequencing of the mutated region.

Fisher exact tests were used to compare the disease incidence, i.e., the percentage of diseased plants, observed with the challenged samples and the corresponding wild-type control.

### Sequencing

Full-length sequencing of the viral genome was performed by Illumina sequencing. For each sequenced Tog7291 infected sample, the inoculum was also sequenced for use as a wild-type control. Total RNA was extracted with the GeneJET Plant RNA Purification kit (Thermo Scientific). Specific retrotranscription of the viral genome was performed by PrimeScript^TM^ Reverse Transcriptase (Takara Bio Inc.) using the 5′-CTCCCCCACCCATCCCGAGAATT-3′ primer (Fargette et al., 2004). Two overlapping fragments covering the complete RYMV genome were amplified by ExTaq polymerase (Takara Bio Inc.) using primers 5′-CAATTGAAGCTAGGAAAGGAG-3′ and 5′-ACTTCGCCGGTTTCGCAGAGGATT-3′ for a first fragment and primers 5′-CATGCTGGGAAAAGTGTCTG-3′ and 5′-CTCCCCCACCCATCCCGAGAATT-3′ for a second fragment (Fargette et al., 2004). The two fragments were equimolary pooled. Libraries were constructed according to the protocol of Mariac et al. (2014) using 6-bp barcodes for multiplexing. DNA was sheared using a Covaris E220 sonicator (Covaris) to reach a mean target size of 400 bp. A paired end sequencing (2 x 150 pb) was performed on a Illumina MiSeq v3 platform at CIRAD facilities (Montpellier, France) with about 12 pmol of the DNA libraries deposited on the flowcell. Technical repeats were performed from RNA of three biological samples, corresponding to RB variants obtained with CIa isolates.

For some Tog7291 infected samples, partial sequencing of the viral genome was performed by Sanger. Retrotranscription was performed with ImProm II Retrotranscripase (Promega) and the primer 5′-ACTTCGCCGGTTTCGCAGAGGATT-3′ (Hébrard et al., 2006). Amplification was performed with GoTaq G2 Polymerase (Promega), using the primers 5′-GGTCGCTTTCTCACTCGCACC-3′ and 5′-GAGAGCCCGACCACCCACTG-3′ (Hébrard et al., 2006). Amplified products were sent to Beckman Coulter Genomics for sequencing with primer 5′-ACCCCAGGATTTACTCTTT-3′.

### Bioinformatics Analysis

Reads were demultiplexed according to their TAG using a free program developed at UMR AGAP, INRA, Montpellier, France^1^. Analysis of the Illumina sequencing data was conducted with the TOGGLEv0.3 suite^2^ (Monat et al., 2015). Ten nucleotides at the 5′-end, two at the 3′-end of each read and adapters were removed with CutAdapt 1.81.1 software (Martin, 2011). A trimming of *q* = 30 and a minimum length of *m* = 35 were applied. Only paired reads were considered for further analysis.

In a first step, the wild-type control sequences were generated by mapping datasets corresponding to the inoculum samples on the reference sequence of an isolate from the same genetic group. The public sequence of the CIa isolate (GenBank AJ608219) from the S2 strain was used as reference for the CIa control dataset, and the public sequence of the Ni1 isolate (GenBank AJ608212) from the S1 strain was used as reference for the control dataset corresponding to Ng106, Ng109, and Tg274 isolates. Mapping was performed with bwaAln and bwaSampe modules of bwa 0.7.12 (Li and Durbin, 2009) using a maximum edit distance of *n* = 8 and default values for additional parameters. Local realignment was performed with the IndelRealigner module of GATK 3.3 (McKenna et al., 2010). The control wild-type sequences in FASTA format were generated from the bam output of IndelRealigner using Geneious 6.0.6^3^ (Kearse et al., 2012). In a second step, all datasets (including control datasets) were mapped on the corresponding wild-type control sequence using the same procedure as previously described, except that a maximum edit distance of *n* = 4 was applied for bwaAln.

SNP calling was performed using LoFreq software (Wilm et al., 2012). Only SNPs with a quality above 200 and a frequency above 5% were retained. SNPs were considered as RB mutations if they are detected in one of the Tog7291 infected samples but not on the corresponding control sample collected on IR64.

To facilitate comparison between datasets corresponding to different isolates, the position of SNPs referred to the position of the corresponding nucleotides in the CIa sequence.

## Results

### Frequency of Tog7291 Resistance Breakdown by RYMV Isolates

**FIGURE 1:**
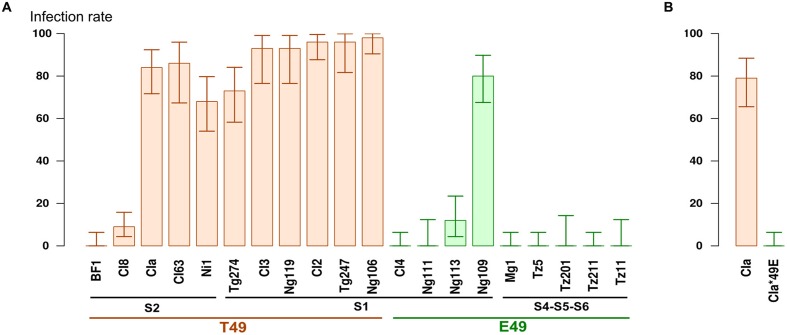
**(A)** Percentage of infected plants on the Tog7291 accession 4 weeks after inoculation. Plants were inoculated with RYMV isolates representing different strains of the virus (S1, S2, S4, S5, S6) and distinguished by a T or E amino acid at position 49 of the VPg. The error bars represent 95% confidence intervals. **(B)** Percentage of infected plants on Tog7291 inoculated with the wild-type CIa isolate and the mutant CIa^∗^49E 4 weeks after inoculation.

We estimated the ability of 20 isolates representative of the diversity of RYMV to infect Tog7291 accession (**Table 1**) based on the inoculation of 28 to 56 plants per isolate. On the IR64 susceptible control, symptoms appeared homogeneously on all plants about 10 days after inoculation. On the Tog7291 accession, symptoms were observed on some plants, but not all, and the symptom onset date ranged from 2 to 6 weeks after inoculation, depending on the plants and isolates, with a heterogeneous pattern compared with the susceptible control. The infection rate was confirmed by ELISA tests performed on individual plants with samples collected 4 and sometimes 6 weeks after inoculation. The infection rate 4 and 6 weeks after inoculation were highly correlated (*R*^2^ > 0.98), and only results obtained 4 weeks after inoculation are presented hereafter. Four weeks after inoculation, the Tog7291 infection rate ranged from 0 to 96% depending on the isolates (**Figure 1**).

We checked if the phenotype observed was linked to incomplete penetrance of the resistance, as observed in some plant/virus interactions (Chandra-Shekara et al., 2006; Ouibrahim et al., 2014), or if the infected plants carried a RYMV variant that had evolved to overcome the resistance. For six isolates (Ng109, Ni1, Tg274, CIa, CI3, CI8), three Tog7291 infected plants were used as inoculum sources for back-inoculation assays on eight to ten Tog7291 plants. The first symptoms appeared 10 days after inoculation. Three weeks after inoculation, the infection rate was significantly higher with inoculum from infected Tog7291 plants than with wild-type isolates for almost all of the tested samples (**Table 2**). This result strongly suggested evolution of the virus to overcome Tog7291 resistance. Consequently, we further considered that the infection cases observed on Tog7291 corresponded to the emergence of RB variants.

**Table 2:** Back-inoculation assays with infected Tog7291 samples.

Isolate	Wild-type	Samples from infected Tog7291
CI3	3/10	10/10^∗∗^
		10/10^∗∗^
		10/10^∗∗^
CI8	2/10	10/10^∗∗^
		10/10^∗∗^
		10/10^∗∗^
CIa	1/8	4/9
		9/9^∗∗^
		9/10^∗^
Ni1	2/10	10/10^∗∗^
		8/8^∗∗^
		9/9^∗∗^
Tg274	2/9	9/9^∗∗^
		11/11^∗∗^
		10/10^∗∗^
Ng109	1/10	11/11^∗∗^
		10/10^∗∗^
		10/10^∗∗^


*Tog7291 accession was inoculated by wild-type isolates multiplied on IR64 or by samples from infected Tog7291 plants. Samples from three different infected Tog7291 plants were tested for each isolate. The ratio between the number of infected plants and the number of inoculated plants was estimated 3 weeks after inoculation. ^∗^ Significant difference between back-inoculation samples and the corresponding wild-type isolates with a *p* < 0.01 threshold (Fisher exact test), ^∗∗^ significant difference with a *p* < 0.0027 threshold (corresponding to a Bonferroni corrected *p* < 0.05 threshold).*

The RB rate of Tog7291 was highly variable between isolates (**Figure 1**). A first set of isolates showing a non-significantly different RB rate contained eight isolates (BF1, CI4, Ng111, Mg1, Tz5, Tz11, Tz201, Tz211) for which there was no detected resistance breakdown, and two isolates, CI8 and Ng113, for which RB rates of 9 and 12% were observed, respectively. RB rates above 68% were obtained for the ten other isolates (CIa, CI2, CI3, CI63, Ni1, Ng106, Ng109, Ng119, Tg247, Tg274), that were thus significantly different from the previous ones. Isolates with a low RB ability belonged to the different strains, but no isolates with a high RB ability were noted among isolates from strains S4, S5, and S6, which originated from East Africa. We analyzed the RB rate regarding E/T polymorphism at position 49 of the VPg, which is associated with the *RYMV1* RB ability (Poulicard et al., 2012). The E49 isolates were found to have a significant lower RB ability (average RB rate of 10%) than T49 isolates (average RB rate of 72%; Fisher exact test: *p* < 0.01).

To confirm the role of polymorphism at position 49 of the VPg on Tog7291 resistance breakdown, we compared the RB rate of the CIa wild-type isolate (T49) with that of the CIa^∗^49E mutant. A significant difference (Fisher exact test: *p* < 0.01) was observed with no RB cases among 56 plants inoculated with the CIa^∗^49E mutant and 44 infected plants out of 56 with the wild-type CIa isolate. This result confirmed the substantial advantage conferred by the T at position 49 of the VPg for Tog7291 resistance breakdown in the CIa genomic background.

### Sequence Analysis of RB Samples

We used Illumina technology to sequence the full-length RYMV genome in seven samples from different Tog7291 plants after inoculation with isolates CIa (three samples), Ng106 (one sample), Ng109 (one sample) and Tg274 (two samples), and the wild-type controls multiplied on IR64.

Between 36788 and 528186 reads of about 140 nucleotides were obtained depending on the samples. More than 94% of the reads were kept after the cleaning step, and between 78 and 92% of them were properly mapped against the references. The average sequencing depth was 5119, but there were marked variations between samples (**Supplementary Table 1**) and along the genome (**Supplementary Figure 1**). In particular, a technical bias increased the sequencing depth at both extremities of the amplified fragments. The minimum sequencing depth was 430. A total of 46 SNPs, with alternate allele frequency ranging from 0.06 to 1, were detected. No differences in SNP detection and few differences in SNP frequencies (<1.1%) were detected between the technical repeats.

Four to six SNPs with alternate allele frequencies ranging from 6 to 40% were detected in the control sequences of CIa, Ng106, and Ng109 isolates. These SNPs were scattered throughout the genome and underlined the heterogeneity of the viral populations (**Supplementary Table 2**). In addition, a fixed and non-synonymous mutation in the coat protein (P32L) was observed in the control CIa sample as compared to the reference sequence of the CIa isolate, suggesting a putative evolution of the isolate since the initial sequence was released.

**FIGURE 2:**
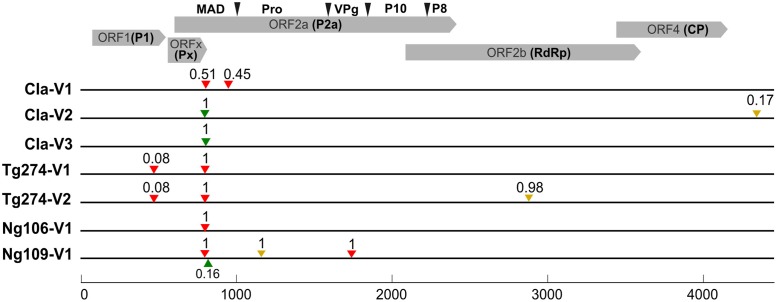
**SNPs observed in RB samples from infected Tog7291 plants.** The virus full-length sequences were obtained by Illumina technology. SNPs were detected by LoFreq after mapping reads on the control sequence of the corresponding isolate. Synonymous SNPs or SNPs in non-coding regions are represented by yellow triangles, non-synonymous SNPs by red triangles, and SNPs that are non-synonymous for polyprotein P2a and synonymous for the Px protein by green triangles. The frequencies of the variants are indicated above the triangles. A nucleotide scale is given in the lower part of the figure. The upper part of the figure indicates the positions of the ORFs and proteins of the RYMV genome. For polyprotein P2a, the positions of the putative cleavage sites between the membrane anchor domain (MAD), the protease (Pro), the VPg and the homologs of SeMV proteins P8 and P10 were determined according to Nair and Savithri (2010).

All samples collected on infected Tog7291 plants presented at least one SNP in the first 115 amino acids of polyprotein P2a, encoded by ORF2a (nucleotidic positions 799, 804, 805, 811 or 951; **Table 3**; **Figure 2**). Based on the putative cleavage sites of sobemovirus polyproteins P2a deduced from the SeMV protease activity (Sõmera and Truve, 2013), this region is supposed to be part of the membrane anchor domain of polyprotein P2a. However, *in silico* analysis suggested that the SNPs observed are not inside the transmembrane segments predicted in the first 60 amino acids (**Supplementary Table 3**). The SNP at position 804 was detected in five RB samples out of seven and also affected the Px protein encoded by ORFx in a non-synonymous way. SNPs at positions 799, 805, and 811 were also located in the overlapping region between ORF2a and ORFx but they were synonymous for the Px protein. The SNP at position 951 only concerned ORF2a. For most of these SNPs, the alternate alleles were fixed (frequency over 98%) in the RB samples, except for the CIa-V1 and Ng109-V1 samples. In the CIa-V1 sample, alternate alleles at positions 804 and 951 had frequencies of about 50%. Phasing analysis suggested that the two mutations were on different haplotypes. In the Ng109-V1 sample, the alternate allele at position 811 had a frequency of 16%, but a fixed SNP was also observed at position 804.

**Table 3:** Non-synonymous mutations observed in the membrane anchor domain of polyprotein P2a.

Nucleotide modification	Amino acid modification	Sample sequenced by Illumina	Sample sequenced by Sanger
A799G	E64G	CIa-V2	
T804C	F66L	Ng106-V1, Tg274-V1, Tg274-V2	CI8 (2 var.), CI2 (2 var.), Ni1 (1 var.)
T804C + T951C	F66L + F115L	CIa-V1	
T804C + G811A	F66L + R68Q	Ng109-V1	
T805G	F66C	CIa-V3	Ni1 (1 var.)
T817C	V70A		CIa (2 var.)


*The nucleotide position refers to the position of the corresponding nucleotide in the genomic CIa sequence. The amino acid modification refers to the position of the amino acid on polyprotein P2a of the CIa isolate. Sample names are indicated for variants sequenced using Illumina technology and only the number of samples is indicated for samples sequenced by Sanger. The mutation at nucleotide position 804 also induces an L to P amino acid change in the protein Px encoded by ORFx.*

In four RB samples, additional SNPs were detected outside the membrane anchor domain of polyprotein P2a. Synonymous SNPs were observed at positions 1166 (in the protease coding region), 2884 (polymerase) and 4346 (3′-UTR) in samples Ng109-V1, Tg274-V2 and CIa-V2, respectively. An SNP at position 1741 changed a histidine (H) into a tyrosine (Y) at position 52 of the VPg in sample Ng109-V1. Interestingly, this mutation had already been described in the resistance breakdown of the *RYMV1* gene (Pinel-Galzi et al., 2007; Traoré et al., 2010; Poulicard et al., 2012). Coding SNPs in the 440–453 interval in the ORF1 encoding the P1 protein were observed at low frequency (8%) in the two Tg274 samples. However, no SNP were considered significant in this region in the control Tg274 sequence due to low alternate allele frequency, but they were still identified by LoFreq software with high quality scores, which suggested that the SNPs detected in Tg274 samples may be false positive, i.e., non-specific to RB variants.

As all the samples sequenced by Illumina showed a non-synonymous SNP in the 799–951 interval, this region was analyzed, via Sanger sequencing, on eight additional samples from infected Tog7291 plants inoculated with isolates CIa, Ni1, CI2, and CI8 (**Table 3**). Six samples showed the mutations already observed at positions 804 and 805. Two samples obtained after inoculation of the CIa isolate presented a mutation at position 817. This mutation induces an amino acid change in the membrane anchor domain of polyprotein P2a and is synonymous for the Px protein.

Finally, in all 15 samples analyzed, the RYMV genome showed amino acid changes in the polyprotein P2a region located upstream from the putative cleavage site between the membrane anchor domain and the protease. This result strongly suggested that the membrane anchor domain is involved in Tog7291 resistance breakdown.

### Validation of the Role of the Mutations in Resistance Breakdown and Analysis of Their Fitness

To confirm the role of the mutations at positions 799, 804, 805, 817, and 951 in Tog7291 resistance breakdown, they were inserted in the CIa infectious clone by directed mutagenesis. Mutants CIa^∗^P2a-64G, CIa^∗^P2a-66C, CIa^∗^P2a-66L, CIa^∗^P2a-70A, CIa^∗^P2a-115L were obtained. The mutants were inoculated on Tog7291 and IR64 after *in vitro* transcription and multiplication on the susceptible *O. sativa* IR64 variety. The infectivity was evaluated 2 weeks after inoculation. On Tog7291, significant differences of infection rate were observed between the mutants and the wild-type CIa isolate for all mutants except the CIa^∗^P2a-64G mutant (**Table 4**). Sanger sequencing of two samples per mutant, collected on Tog7291, indicated that the mutations introduced by directed mutagenesis were stable. Those results indicated that mutations F66C, F66L, V70A, and F115L were involved in resistance breakdown in the Tog7291 accession and would be sufficient for resistance breakdown in the CIa genetic background. Our results did not allow us to draw any conclusions regarding the E64G mutation. On the susceptible variety IR64, the infection rate observed after inoculation of the mutants was low and significantly different from the infection rate observed after inoculation with the wild-type isolate. This observation strongly suggested a loss of fitness of the mutants on IR64.

**Table 4:** Infection rate on Tog7291 and IR64 after inoculation of mutants obtained by directed-mutagenesis and a single multiplication step on IR64.

RYMV genotype	IR64	Tog7291
CIa	8/8	2/21
CIa^∗^P2a-64G	0/8^∗^	7/21
CIa^∗^P2a-66C	1/8^∗^	27/27^∗^
CIa^∗^P2a-66L	2/7^∗^	18/28^∗^
CIa^∗^P2a-70A	0/5^∗^	28/28^∗^
CIa^∗^P2a-115L	0/8^∗^	22/26^∗^


*The CIa wild-type isolate was inoculated as a control. The ratio between the number of infected plants and the number of inoculated plants 2 weeks after inoculation is indicated. ^∗^Significant difference between the mutant and the CIa wild-type isolate with a *p* < 0.01 threshold (Fisher exact test).*

After a second multiplication of the mutants on IR64, the inoculum concentration was adjusted by Q-RT-PCR and the variants were inoculated on IR64, Tog7291 and the susceptible *O. glaberrima* Tog5673 accession. On IR64, almost all inoculated plants were infected regardless of the inoculum (**Table 5**), contrary to the results obtained previously (**Table 4**). However, sequencing of three samples per variant revealed that the mutants had reverted to the wild-type CIa genotype in half of the plants. On Tog5673, the infection rate was similar after inoculation of the mutant and the control and only the CIa^∗^P2a-66L variant had reverted. On Tog7291, the infection rate was lower than that observed after a single multiplication step on IR64, but all the sequenced samples had conserved the mutations. These results revealed a progressive reversion of the mutations after inoculation on IR64 and supported a marked loss of fitness in RB variants on IR64. The presence of the mutated genotype on Tog5673 and Tog7291 suggested that, after two multiplication steps on IR64, the reversion process was incomplete, and that the mutants’ loss of fitness was lower in Tog5673 than in IR64. While incomplete, this reversion process probably explained why the infection rate on Tog7291 was lower than in the previous experiment.

**Table 5:** Fitness of mutants obtained by directed mutagenesis and two successive multiplications on IR64.

Starting genotype	IR64		Tog5673		Tog7291	
			
	Number of infected plants	Final genotype	Number of infected plants	Final genotype	Number of infected plants	Final genotype
CIa^a^	5		5		1	
CIa^b^	5		5		1	
CIa^∗^P2a-64G^a^	4	1 st., 2 rev.	5	2 st.	2	2 st.
CIa^∗^P2a-70A^a^	5	2 st., 1 rev.	5	1 st.	2	2 st.
CIa^∗^P2a-66C^b^	5	1 st., 2 rev.	5	2 st.	5	3 st.
CIa^∗^P2a-66L^b^	5	3 rev.	5	3 rev.	4	3 st.
CIa^∗^P2a-115L^b^	5	3 st.	5	3 st.	2	2 st.


*Five plants of each accessions were inoculated with each mutant. The number of infected plants was determined by ELISA test 2 weeks after inoculation. The number of sequenced samples in which the mutation was stable (st.) or had reverted to the wild-type genotype (rev.) is indicated. ^a^The inoculum was adjusted to about 2.10^9^ copies per plant, ^b^The inoculum was adjusted to about 3.10^10^ copies per plant.*

## Discussion

In this study, we reported infection events by RYMV on Tog7291, an *O. glaberrima* accession previously described as highly resistant to that virus (Thiémélé et al., 2010; Orjuela et al., 2013). In plant/virus interactions, incomplete penetrance of resistance has been described in several cases and could explain why a plant with a resistant genotype can develop the disease (Ouibrahim et al., 2014; Poque et al., 2015). Penetrance may be dependent on the environmental conditions, such as temperature or light (Collmer et al., 2000; Chandra-Shekara et al., 2006). However, in our study, a high infection rate after back-inoculation assays on Tog7291 and the identification of mutations in the sequence of the virus from infected samples indicated that Tog7291 infection was associated with the emergence of RB variants.

*Rice yellow mottle virus* isolates showed a highly contrasted ability to evolve toward virulence, according to the virulence definition given by Vanderplank (1968), i.e., the qualitative capacity of a parasite to infect a host. Ten isolates overcame Tog7291 resistance in less than 13% of the inoculated plants, while 10 others overcame this resistance in more than 60% of the plants. The ability to overcome Tog7291 resistance was significantly associated with the T/E polymorphism at amino acid 49 of the VPg. The role of this polymorphism was confirmed by the comparison of the wild-type CIa isolate (T49) and its CIa^∗^49E mutant obtained by directed mutagenesis. Similarly, T/E polymorphism is a key genetic determinant of the ability to overcome Gigante (*O. sativa*) and Tog5681 (*O. glaberrima*), two accessions possessing different resistance alleles on the *RYMV1* gene (Poulicard et al., 2012): T49 isolates frequently overcome resistance of Tog5681 and rarely that of Gigante, while opposite results have been obtained for E49 isolates. In addition, studies on Tog5307, an *O. glaberrima* accession whose resistance is associated with the *RYMV3* gene, which is supposed to be a classical NB-LRR resistance gene, revealed the same pattern of interaction with T49 and E49 isolates (Pidon et al., submitted). According to Poulicard et al. (2012), T49 isolates had a marked selective advantage over E49 isolates in susceptible *O. glaberrima* accessions when T49 and E49 isolates were inoculated in competition. The impact of trade-offs among different host species and at different steps of the viral cycle have been documented for several plant viruses (Wallis et al., 2007; Agudelo-Romero et al., 2008; Elena et al., 2009) and we showed here that these trade-offs also have an impact on the resistance breakdown of independent resistance genes. The best fitness noted in the *O. glaberrima* genetic background would probably explain the higher capacity of T49 isolates to evolve and acquire RB mutations on resistant *O. glaberrima* accessions, irrespective of the resistance genes. To gain further insight into the interaction between RYMV isolates, resistance genes and genetic background, we are currently investigating the pattern of resistance breakdown specificity by T49 and E49 isolates using near isogenic lines of IR64 (*O. sativ*a spp. *indica*) introgressed with different resistance alleles from *O. glaberrima*. Besides, regardless of the role of T/E polymorphism, other viral genetic determinants are supposed to be involved in the RB ability, as highlighted by the fact that isolates BF1 and CI8 (T49) seldom overcame Tog7291 resistance, or isolate Ng109 (E49) that frequently overcame it.

The full RYMV genome sequencing in infected Tog7291 samples using the Illumina technique allowed rapid and inexpensive identification of RB associated mutations. Next generation sequencing techniques previously proved to be a powerful method to analyze the evolution or the structure of a viral population (Fabre et al., 2012a; Sorel et al., 2014). Here, this method was particularly interesting as no hypothesis on the viral genes involved in resistance breakdown were available and, contrary to Sanger sequencing, allowed the detection of mutations that were not fixed in the viral population.

In all the sequenced samples collected on infected Tog7291 plants, the virus acquired a non-synonymous mutation at the beginning of the polyprotein P2a sequence, and the role of four different mutations in resistance breakdown was validated by directed mutagenesis. The mutation at position 804, which changes a phenylalanine into a leucine in polyprotein P2a, was observed in eight samples out of 15, and was by far the most frequent mutation. Contrary to the other mutations, this mutation also affected the Px protein encoded by ORFx in a non-synonymous way. ORFx was recently identified by computational analysis (Ling et al., 2013) and the role of the encoded Px protein is still unknown. Nevertheless, as seven RB samples did not show any amino acid substitution in the Px protein, we supposed that the resistance breakdown was likely due to mutations in polyprotein P2a. In SeMV, polyprotein P2a is cleaved by *cis*- or *trans*-proteolytic activity in the membrane anchor domain, the serine protease, the VPg, the P10 and the P8 proteins (Nair and Savithri, 2010). The membrane anchor domain of SeMV consists of the first 132 amino acids of polyprotein P2a. *In silico* research of putative cleavage sites in the RYMV polyprotein P2a (Sõmera and Truve, 2013) suggested that RB mutations occurred in this domain. This domain is believed to anchor the polyprotein into cellular membranes to facilitate proteolytic processing and probably also to be involved in viral minus strand synthesis (Sõmera et al., 2015). Noteworthy, the positions mutated in polyprotein P2a of RB variants are conserved in RYMV diversity and the mutations detected have never been observed before.

A mutation at position 52 of the VPg (H52Y) was observed, together with the mutation F66L, in an RB sample obtained after inoculation with isolate Ng109. Interestingly, this mutation was frequently observed in *rymv1-3* resistance breakdown in Tog5681 accession (Traoré et al., 2010). Its effect on Tog7291 resistance breakdown will have to be confirmed. Alternatively, as this mutation appeared in one of only two E49 isolates able to overcome Tog7291 resistance, it may be involved in the fitness of E49 isolates in *O. glaberrima* accessions, while F66L mutation is directly responsible of resistance breakdown.

In Tog7291, resistance is associated with a truncated allele of the gene *RYMV2*, also named *CPR5-1*, leading to a non-functional protein (Orjuela et al., 2013). In *A. thaliana*, the *CPR5* gene is involved in control of the cellular cycle and defense mechanisms (Bowling et al., 1997; Kirik et al., 2001; Wang et al., 2014). According to Gu et al. (2016), CPR5 is a transmembrane nucleoporin that undergoes a oligomer to monomer conformational switch in response to the activation of nucleotide-binding leucine-rich repeat receptors. This conformational switch triggers the release of cyclin-dependent kinase inhibitors and the permeabilization of the nuclear pore complex that lead to effector triggered immunity. In the rice/RYMV interaction, the role of *CPR5* and the role of RB mutations on *CPR5* mediated resistance are still unknown, but different hypotheses could be considered. As observed in *A. thaliana*, the presence of a null allele of *CPR5-1* in Tog7291 may trigger the activation of defense mechanisms, resulting in high resistance to RYMV. RB mutations could modify the susceptibility of RYMV to one of this defense mechanisms, which would result in a less efficient resistance. Alternatively, an interaction between the polyprotein P2a and CPR5-1 may be required for the virus cycle. Tog7291 resistance would result from a defective interaction, as observed for resistance mediated by translation initiation factors (Sanfaçon, 2015). Two paralogs of *CPR5* are present on the rice genome, the RB mutations could allow a functional interaction between the polyprotein P2a and the protein encoded by *CPR5-2*, paralog of *CPR5-1*. Similarly, translation initiation factors belong to small multigenic families and *Turnip mosaic virus* is able to interact with different members of these families (Jenner et al., 2010), even though resistance breakdown does not obligatory involve a switch from one member to another (Gallois et al., 2010).

A resistance breakdown fitness cost has been described in several plant/virus interactions involving both major resistance genes (Garcia-Arenal and Fraile, 2013, for review) and quantitative resistance (Montarry et al., 2012). Antagonistic pleiotropy of RB mutations frequently increases the fitness in one host but reduce within-host multiplication in another (Jenner et al., 2002; Poulicard et al., 2010; Moreno-Pérez et al., 2016). Besides, RB mutations may also affect the stability (Hamada et al., 2007) or the transmission of viruses, and these independent deleterious effects of RB mutations may explain why some resistance genes are not overcome in the field even though RB mutants have been observed in experimental conditions (Sorel et al., 2014). In this study, we challenged mutants virulent on Tog7291 on two susceptible accessions. On the IR64 susceptible accession (*O. sativa*), the low disease infectivity or the frequent reversion to the wild-type genotype suggested a fitness cost of RB mutations. However, the lower number of reversions on Tog5673 implied that the fitness cost of resistance breakdown may be host-dependent. As Tog5673 and Tog7291 are both *O. glaberrima* accessions, while IR64 is an *O. sativa indica* variety, it could be interesting to test whether the fitness penalty depends on the host species. As for the T/E polymorphism at position 49 of the VPg, such a host species/RYMV variant interaction would greatly impact the durability of resistance. Besides, it is worth noting that the only mutation that has reverted in Tog5673 is that at nucleotidic position 804, which affects both the Px protein and polyprotein P2a. While most frequently observed on Tog7291, this mutation appeared to be associated with a marked loss of fitness, whatever the host genotype. This may result from its pleiotropic effect on two different proteins, in agreement with the assumption that, in plant/virus interactions, the cost of resistance breakdown is enhanced by the small size of the viral genome, which is constrained by multifunctional proteins and overlapping genes (for review, Sacristan and Garcia-Arenal, 2008).

Finally, our study highlighted different trade-offs acting in host range evolution of plant viruses, as mentioned by Garcia-Arenal and Fraile (2013). The E/T polymorphism in the VPg is associated to trade-offs among host species and impact both the multiplication of the virus in a susceptible host (Poulicard et al., 2012) and the frequency of breakdown in a resistant host (Traoré et al., 2010; this study). The RB mutations are associated to trade-offs among genotypes, and also impact the fitness on normally susceptible plants (Poulicard et al., 2010; this study). It is worth noting the complex interplay between both, as some RB mutations, but not all, potentially have differential effects on susceptible genotypes depending on the host species. These results illustrated how the balance between different host species, as well as the balance between different resistant varieties, can impact the durability of resistance genes and are worth being considered to achieve sustainable resistance.

## Author Contributions

AP-G, EH, AG, and LA designed the experiments, LA and AP-G conducted the phenotyping, AP-G, EH, CM, and LA performed the molecular experiments, CD-T and LA performed the bioinformatic analysis, EH and AP-G provided and characterized the viral material, LA wrote the paper, and all the authors revised the paper and approved the version to be published.

## Conflict of Interest Statement

The authors declare that the research was conducted in the absence of any commercial or financial relationships that could be construed as a potential conflict of interest.

## Footnotes

1 https://github.com/Maillol/demultadapt

2 https://github.com/SouthGreenPlatform/TOGGLE

3 http://www.geneious.com
