# Supplementary material for: Mutations in Rice yellow mottle virus Polyprotein P2a Involved in RYMV2 Gene Resistance Breakdown
Source: Front Plant Sci. 2016 Nov 28;7:1779. doi: 10.3389/fpls.2016.01779 (PMC5125353; doi:10.3389/fpls.2016.01779)
Supplement: Supplementary file 1 [file Table_1.PDF]

Supplementary Table 1

Mutations in *Rice yellow mottle virus* polyprotein P2a involved in *RYMV2* gene resistance breakdown

Agnès Pinel-Galzi[1], Christine Dubreuil-Tranchant[2], Eugénie Hébrard[1], Cédric Mariac[2], Alain Ghesquière[2], Laurence Albar[2*]

* Correspondence: Laurence Albar laurence.albar@ird.fr

Supplementary Table 1. Depth of coverage for samples sequenced by Illumina methodology.

Sample	Mean	Minimum	Maximum
Cla-WT	3946	2226	15216
Cla-V1 (tech. rep. 1)	14426	5891	55356
Cla-V2 (tech. rep. 1)	6759	3327	27532
Cla-V3 (tech. rep. 1)	9074	3259	42453
Cla-V1 (tech. rep. 2)	1885	791	8281
Cla-V2 (tech. rep. 2)	1760	580	7802
Cla-V3 (tech. rep. 2)	2258	926	10052
Ng106-WT	2889	1509	12563
Ng106-V1	1272	658	4647
Ng109-WT	1628	988	5404
Ng109-V1	9779	3416	38653
Tg274-WT	10570	6003	36354
Tg274-V1	4444	2586	16116
Tg274-V2	980	430	3910
**Mean**	**5119**	**2333**	**19244**
