# Supplementary material for: Mutations in Rice yellow mottle virus Polyprotein P2a Involved in RYMV2 Gene Resistance Breakdown
Source: Front Plant Sci. 2016 Nov 28;7:1779. doi: 10.3389/fpls.2016.01779 (PMC5125353; doi:10.3389/fpls.2016.01779)
Supplement: Supplementary file 3 [file Table_3.pdf]

Supplementary Table 3

Mutations in *Rice yellow mottle virus* polyprotein P2a involved in *RYMV2* gene resistance breakdown

Agnès Pinel-Galzi[1], Christine Dubreuil-Tranchant[2], Eugénie Hébrard[1], Cédric Mariac[2], Alain Ghesquière[2], Laurence Albar[2*]

* Correspondence: Laurence Albar laurence.albar@ird.fr

Supplementary Table 3. Predicted transmembrane segments in the polyproteins P2a of RYMV and SeMV.

Predictors	SeMV	RYMV
DAS-TM[a]	13-24, 39-62	24-36, 51-53
TMPred	8-29, 36-66	22-47, 44-60
TMHMM	10-32, 39-61	-
HMMTOP	9-30, 39-61[b]	21-37, 42-60
Phobius	6-30, 36-61	21-37, 43-60

[a] Predictor used in Satheshkumar, P. S., Lokesh, G. L., and Savithri, H. S. (2004). Polyprotein processing: Cis and trans proteolytic activities of *Sesbania mosaic virus* serine protease. *Virology* 318, 429–438. doi:10.1016/j.virol.2003.09.035.

[b] A third TM domain was predicted at the C-terminus of SeMV P2a (aa 737-753).
