# Supplementary material for: Mutations in Rice yellow mottle virus Polyprotein P2a Involved in RYMV2 Gene Resistance Breakdown
Source: Front Plant Sci. 2016 Nov 28;7:1779. doi: 10.3389/fpls.2016.01779 (PMC5125353; doi:10.3389/fpls.2016.01779)
Supplement: Supplementary file 4 [file Image_1.PDF]

Supplementary Figure 1

Mutations in *Rice yellow mottle virus* polyprotein P2a involved in *RYMV2* gene resistance breakdown

Agnès Pinel-Galzi[1], Christine Dubreuil-Tranchant[2], Eugénie Hébrard[1], Cédric Mariac[2], Alain Ghesquière[2], Laurence Albar[2*]

* Correspondence: Laurence Albar laurence.albar@ird.fr

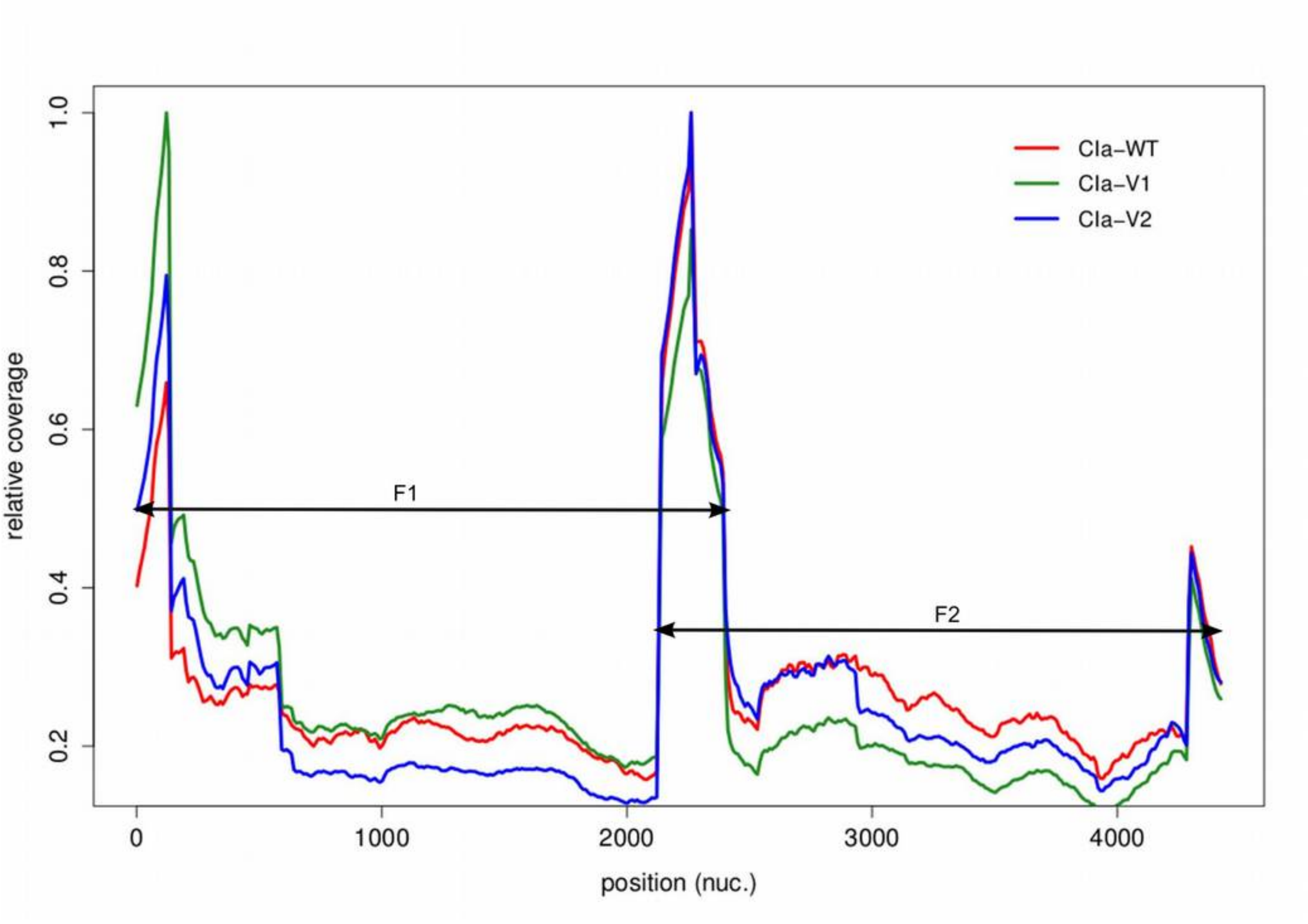

Supplementary Figure 1. Normalized depth of coverage along the genome for the control CIa sample (CIa-WT) and the infected Tog7291 samples CIa-V1 and CIa-V2. Normalization was performed by dividing the coverage at each nucleotide by the maximum coverage of the sample. A higher sequencing depth is observed at both extremities of the amplified fragments, F1 and F2, represented by black arrows.
